# Potential roles of N6-methyladenosine (m6A) in immune cells

**DOI:** 10.1186/s12967-021-02918-y

**Published:** 2021-06-08

**Authors:** Chang Liu, Zhe Yang, Rong Li, Yanju Wu, Ming Chi, Shuting Gao, Xun Sun, Xin Meng, Biao Wang

**Affiliations:** 1 Department of Radiation Oncology, The First Affiliated Hospital of China Medical University, No. 155 NanJing North Road, Shenyang, China; 2 College of Life Science, Liaoning University, 66 Chongshan Road, Shenyang, 110036 People’s Republic of China; 3 Department of Biochemistry and Molecular Biology, School of Life Sciences, China Medical University, No. 77 Puhe Road, Shenyang North New Area, Shenyang, 110122 Liaoning China; 4 Department of Immunology, College of Basic Medical Sciences of China Medical University, , No. 77 Puhe Road, Shenyang North New Area, Shenyang, 110122 Liaoning China

**Keywords:** N6-methyladenosine, Immune cells, RNAs

## Abstract

N6-methyl-adenosine (m6A) is one of the most common internal modifications on RNA molecules present in mammalian cells. Deregulation of m6A modification has been recently implicated in many types of human diseases. Therefore, m6A modification has become a research hotspot for its potential therapeutic applications in the treatment of various diseases. The immune system mostly involves different types of immune cells to provide the first line of defense against infections. The immunoregulatory network that orchestrate the immune responses to new pathogens plays a pivotal role in the development of the disease. And m6A modification has been demonstrated to be a major post-transcriptional regulator of immune responses in cells. In this review, we summarize the participants involved in m6A regulation and try to reveal how m6A modification affects the immune responses via changing the immunoregulatory networks.

## Introduction

The epigenetic regulation of gene expression plays a vital role in a wide range of physiological processes and pathological events, which has been considered as one of the most critical research areas for optimal control of gene expression. A large number of studies have shown that the aberrant epigenetic changes are associated with a wide range of diseases, including cancers, diabetes, Alzheimer’s disease and other age-related diseases [1]. In the last few decades, there was an explosion in epigenetics research, focusing on DNA modifications. And RNA modifications are relatively young players in the field of epigenetic regulation [2], which has gained an increasing attention in recent years. So far, more than 100 different types of RNA modifications have been identified on messenger RNA (mRNA), ribosomal RNA (rRNA), transfer RNA (tRNA), small nuclear RNA (snRNA), microRNA (miRNA), and long non-coding RNA (lncRNA) [3, 4] from viruses, yeast, and mammals [5, 6]. Among them, RNA N6-methyladenosine (m6A) modification, a well-known modification first discovered 1974, has been regarded as the most prevalent internal mRNA modification in mammalian cells [7, 8]. Although it was identified many decades ago, its biological roles and mechanism of m6A modification in human diseases remain unclear. Recently, with the development of m6A detection methods, more and more biological functions and its potential significance [2] are being revealed and elucidated [9]. It is reported that m6A can regulate alternative splicing of pre-mRNAs [10], cap-independent translation [11], mRNA stability [12] and miRNA biogenesis [13]. Besides these, m6A is also involved in the regulation of various biological processes, such as stem cell differentiation [14], cellular reprogramming [15], heat shock response [16], circadian adjustment [17] and cancer development [18].

The human immune system is a kind of defense weapon to protect our bodies against attacks from disease-causing pathogens, using several different effector mechanisms to recognize and destroy the broad range of pathogens that it encounters. It is a complicated and dynamic network of cells and molecules [19] that enables rapid responses to environmental stimuli. But the disorders of the immune system can lead to recurrent infections, autoimmunity, malignancies, hematological disorders, degenerative diseases, and many other problems [20]. An effective immune response against invading pathogens relies on the cooperation between different types of immune cells. Therefore, it’s most important to find a new molecular pathway that controls the collaboration between immune cells for the better understanding on the role of immunoregulation in human disease development.

m6A modification as a new layer of regulatory mechanism in controlling gene expression plays vital roles in regulating various aspects of immunity, surely becoming the research focus in the coming future. In this review, we summarize the recent findings on how m6A works in different types of immune cells.

## m6A methylation related proteins

**Fig. 1:**
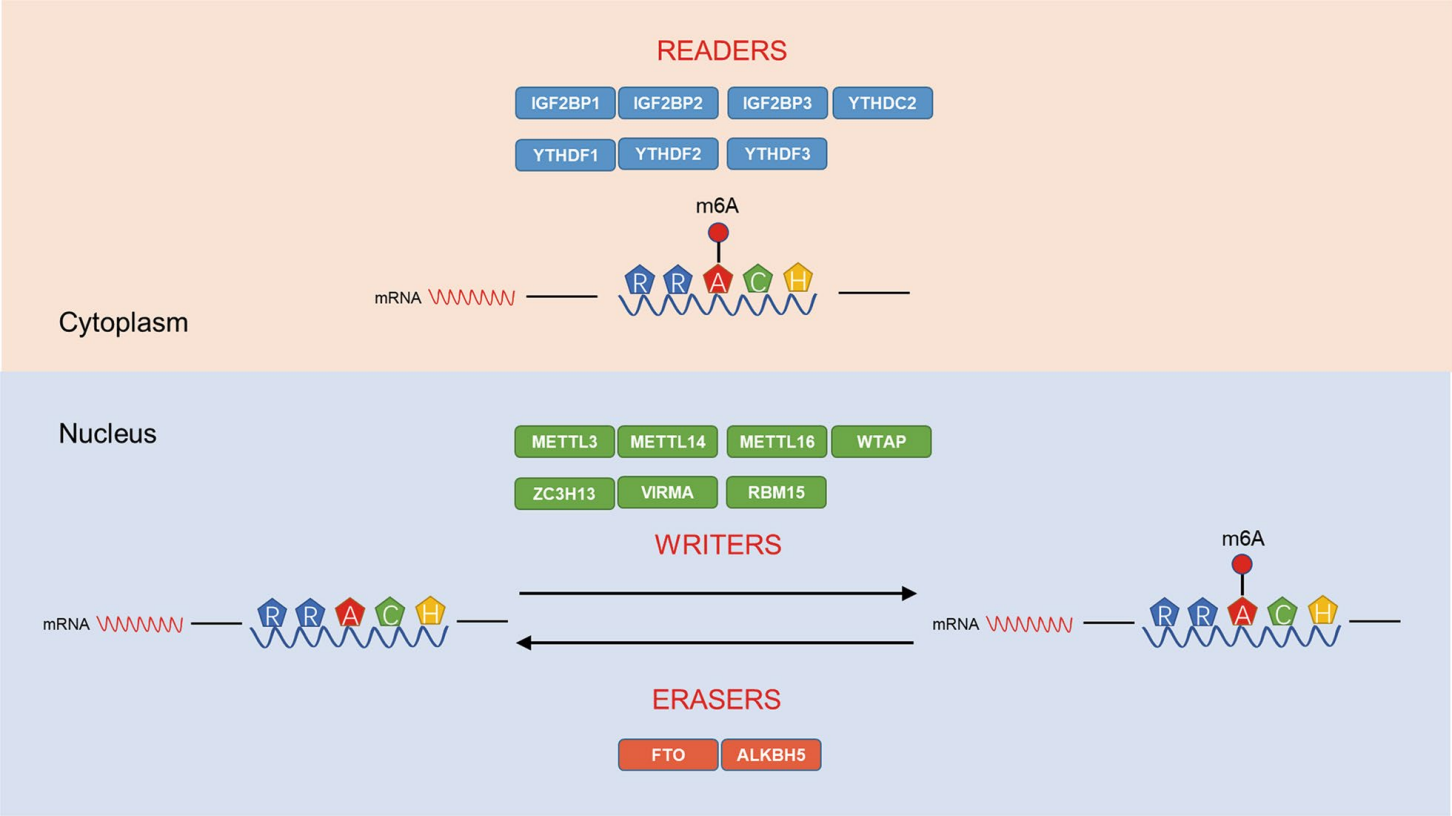
m6A modification related proteins and their localizations in cells. The m6A modification is catalyzed by the writer proteins including METTL3, METTL14, METTL16, WTAP, ZC3H13, VIRMA and RBM15. And the eraser proteins FTO or ALKBH5 removes m6A modification. The m6A reader proteins, including IGF2BP1, IGF2BP2, IGF2BP3, YTHDF1, YTHDF2, YTHDF3 and YTHDC2, recognize m6A and determine the fates of m6A-modified RNAs

The discovery of proteins involved in m6A regulation has greatly promoted the researches of this modification, elucidating their roles as “writers” (m6A methyltransferases), “erasers” (m6A dimethyl transferases), and “readers” (effectors recognizing m6A) (Fig. 1).

### Writers

m6A is installed to mRNA via methyltransferase complex (MTC) that consists of METTL3 catalytic subunit and other accessory subunits, including METTL14, WTAP, Virma, RBM15 and ZC3H13 [21]. METTLE3 was first discovered in 1997 and it is the 70 kDa subunit of MT-A with a SAM-binding domain and a DPPW motif (Asp-Pro-Pro-Trp) [22]. The localization pattern of METTL3 is of diversity in response to cellular stresses. In nucleus, METTL3 interacts with METTL14 to form a stable heterodimer in the ratio of 1:1, facilitating the recognition of target RNAs. It was reported that the nuclear localization signals (NLS) have been identified in both METTL3 and WTAP [23], mediating nuclear import of WTAP and the METTL3/14 heterodimer. In addition, some different components that interact with METTL3 have also been found in the cytoplasm of human cancer cell lines, including HeLa cells [24], breast cancer cells (MDA-MB-231 cells) and acute myeloid leukemia cells [25].

Knockout of METTL3 gene leads to reduction of m6A levels in human HeLa and HepG2 cells, accompanied by apoptosis [26]. In gastric cancer, the up-regulated METTL3 promotes EMT process in vitro and metastasis in vivo via enhancing the stability of ZMYM1 [24]. METTL3 regulates self-renewal of hematopoietic stem cells (HSCs) by enhancing the expression of self-renewal related genes, such as Nr4a2, p21, Bmi-1 and Prdm1 [27]. In human non-small cell lung cancer, METTL3 which undergoes sumoylation has a reduced ability to catalyze m6A, leading to enhanced tumorigenesis [28, 29]. In the case of DNA UV damage, METTL3/14 localized to the sites of UV-induced damage within 2 min with the enhancement of m6A intensity [30]. METTL3 can enhance the m6A modification on histone methyltransferase Ezh2 to promote its expression affecting the neurogenesis and development [31]. Different from the nuclear METTL3, cytoplasmic METTL3 can identify m6A independent of its catalytic activity. In the cytoplasm of lung cancer cells, METTL3 enhances translation efficiency of mRNA by recognizing the m6A in 3′UTR based on the interaction between METTL3 and eIF3h which are co-overexpressed in many types of cancer [24]. As the other core m6A writer, METTL14 contains two conserved functional domains, a SAM-binding domain that participates in catalyzing methylation reactions, an EPPL motif (Glu-ProPro-Leu), an additional protein–protein interacting N-terminal coiled domain and a G-rich sequence at the C-terminus [32]. METTL14 plays a primary role in the recognition of RNA substrates in mammalian cells. The METTL3/14 heterodimer preferentially binds to the RNA substrate that contains the GGACU sequence [14]. In Epstein-Barr virus (EBV) latently infected cells, the expression of METTL14 was significantly increased and stabilized by the EVB antigen EBNA3C, inducing m6A methylation on a number of tumor-suppressor genes and thus increasing the probability of malignancy. METTL14 mediates the self-renewal ability of hematopoietic stem cells by increasing the expression of self-renewal related genes, such as Bmi-1 and Prdm [27]. And METTL14 regulates the development of post-implantation embryos by promoting the transformation of ectodermal cells from a naive state to a priming state. Deletion of METTL14 gene impairs the ability of initiation and differentiation in embryonic stem cells [33]. Other key components of the MTC, such as WTAP and Virma, are required for maintaining the MTC activity and play a regulatory role in the complex [26]. WTAP is a splicing factor that interacts with METTL3/14 heterodimer, which is critical for the localization of nuclear speckles and required for m6A methylation activation. WTAP can also recruit other related factors to the methyltransferase complex, regulating the methylation activity.

METTL16 is also a methyltransferase that can function alone and catalyze m6A of mRNAs, lncRNAs and U6 small nuclear RNA (U6 snRNA) [34], which requires the UACAGAGAA sequence. And the N-terminal module of METTL16 is essential for RNA binding [35]. The interaction between METTL16 and MAT2A HP1 promotes splicing of correct intron to adjust the homeostasis of S-adenosylmethionine (SAM) [36] in HEK293 cells.

In Drosophila, RNA-binding protein RBM15 and its homologue RBM15b interact with METTL3 via WTAP and the absence of RBM15/RBM15b will result in the most prominent decrease of m6A deposition [37, 38]. The domains of RBM15/RBM15b preferentially bind to U-rich regions of RNA, recruiting the m6A complex to sites proximal to the m6A consensus motifs [38]. In addition, CBLL1, an E3 ubiquitin ligase, has been identified to have the potential m6A catalytic activity but its role needs to be further studied and clarified [37].

### Erasers

m6A modification in RNAs is a dynamic and reversible process which has been corroborated by the discovery of ‘eraser’ FTO and ALKBH5. And FTO and ALKBH5 are ferrous iron and α-ketoglutarate (αKG) cofactors-dependent demethylases [39], having an AlkB domain in common that consists of two active motifs named HXDXnH and RXXXXXR (X = any amino acid). The differential expression and cellular localization of FTO and ALKBH5 among different tissues reveals that the two proteins have many different and varied biological functions. It is reported that FTO is highly expressed in fat and brain tissues, while ALKBH5 is mainly expressed in the testis. Therefore, demethylation in some organs may be performed only through FTO or ALKBH5 [32]. Studies have shown that FTO preferentially demethylates m6A, thereby affecting mRNA stability in a m6A independent manner [40]. In melanoma, FTO weakens the IFNγ-induced killing effects in vitro via up-regulating PD-1, CXCR4 and SOX10 through inhibiting YTHDF2-mediated-degradation and blocks the response to anti-PD-1 blockade immunotherapy [41]. The elevated expression of FTO promotes the proliferation and metastasis in acute myeloid leukemia (AML) cells. The tumor metabolite 2-hydroxyglutaric acid can prevent disease progression in leukemia by inhibiting the FTO expression [42].

In the area of human cancer research, studies have been carried out trying to reveal the underlying regulatory mechanisms of ALKBH5 in human cancers, but it still remains unclear as well as controversial. In different cancers, ALKBH5 was up-regulated or down-regulated, and played an oncogenic or tumor suppressive role in breast cancer, gastric cancer, and colon cancer [43–45]. The communication between ALKBH5 and long non-coding RNAs, miRNAs or mRNA is closely related with cancer cell proliferation, death, survival, and metastasis [43, 46, 47]. In the field of human non-cancer research, ALKBH5 was involved in human reproductive system diseases via impacting on spermatogenesis and trophoblast invasion [48], or osteogenesis through AKT and NF-κB signaling pathways [49, 50].

### Readers

The readers are required for properly decoding the m6A RNA methylation information in cells, because the secondary or tertiary structure of an m6A-modifed RNA has been changed but without coding-sequence alterations. The YT521-B homology (YTH) domain consists of the YTH domain family 1-3 (YTHDF1-3) and the YTH domain containing protein 1-2 (YTHDC1-2), serving as the module for recognizing m6A [51]. YTHDF1 interacts with translation initiation factor eIF3 to improve the efficiency of cap-dependent translation. And YTHDF1 promotes the degradation of neoantigens in dendritic cells by recognizing the m6A-modified transcripts encoding lysosomal protease, and blocks dendritic cells from delivering tumor neoantigens to T cells, allowing tumor cells to escape from immune surveillance [52]. It is reported that the expression of YTHDF1 is significantly increased in colorectal cancer. Deletion of YTHDF1 gene inhibits the Wnt/β-catenin signaling pathway, thus reducing the tumorigenicity of colorectal cancer cells [52]. YTHDF2 recruits the CCR4-NOT complex through a direct interaction between its N terminus and the SH domain of CNOT1 subunit, and then catalyzes degradation of m(6)A-containing RNAs in mammalian cells [53, 54]. In prostate cancer, the decreased expression of YTHDF2 by miR-493-3p can affect the cell proliferation and migration. YTHDF3 interacts with YTHDF1 and YTHDF2 to not only promote the YTHDF1-facilitated translation of methylated RNA but also increase YTHDF2-mediated mRNA degradation [55]. The direct interaction between eIF4G2 and YTHDF3 mediates initiation of circRNA translation [56].

YTHDC1, the only unique nuclear m6A reader protein, promotes exon inclusion bodies via recruiting SRSF3 while blocking SRSF10 to their targeted RNAs [57]. YTHDC1 can recognize m6A-modified mRNA of MAT2A, therefore, it is required for SAM-responsive regulation of MAT2A [58]. YTHDC2 is present in both the nucleus and the cytoplasm, regulating the stabilization and translation of mRNA during spermatogenesis in mice [59]. It is an RNA-induced ATPase with 3′-5′ RNA helicase activity [60]. A nuclear m6A reader was recently discovered within the pre-mRNA consensus motif RRACH that disrupts the stability of the stem structure and enables the U-tract motif to be more easily exposed to heterogeneous nuclear ribonucleoprotein (HnRNP) binding in a single strand, altering alternative splicing of target RNA [61]. In addition to the above proteins, many other m6A readers have recently been discovered. FMR1 contains 3 KH domains and 1 RRG domain and preferentially binds to m6A-modified RNAs, which may affect RNA translation and stability through the interaction with YTHDF1 and YTHDF2 [62]. mRNA binding protein Insulin-like growth factor 2 mRNA binding protein 1-3 (IGF2BP1-3) is suggested to stabilize target mRNAs in an m6A-dependent manner [63]. IGF2BPs promote the stability of target mRNA by recruiting RNA stabilizers such as ELAVL1 and MATR3 under normal conditions, and promote the storage of target mRNA by shifting to stress particles under stress conditions, enhancing the expression of target mRNA [64]. In HCC, TCAM1P-004 interacts with IGF2BP1 to inhibit the cell proliferation by suppressing the translation of IGF2 and increasing the expression of DDIT3 [64]. IGF2BP3 expression has been significantly upregulated in many cancers, which is an additional predictor of poor prognosis. IGF2BP3 is involved in the fetal-adult hematopoietic switch by interacting with the RNA-binding protein Lin28b. In B cell precursors, Lin28b and IGF2BP3 maintain the stability of mRNAs, such as B cell regulators Pax5 and Arid3a [65]. Nucleoprotein hnRNPA2B1 recruits the complex Dgcr8 into a subset of m6A-modified precursor miRNAs to facilitate their development towards mature miRNAs [13]. FMR1 preferentially recognizes the sequence GGm6ACU via the RGG domain binding and represses translation by stalling ribosomal translocation on the transcripts [66].

## m6A in immune cells

**Fig. 2:**
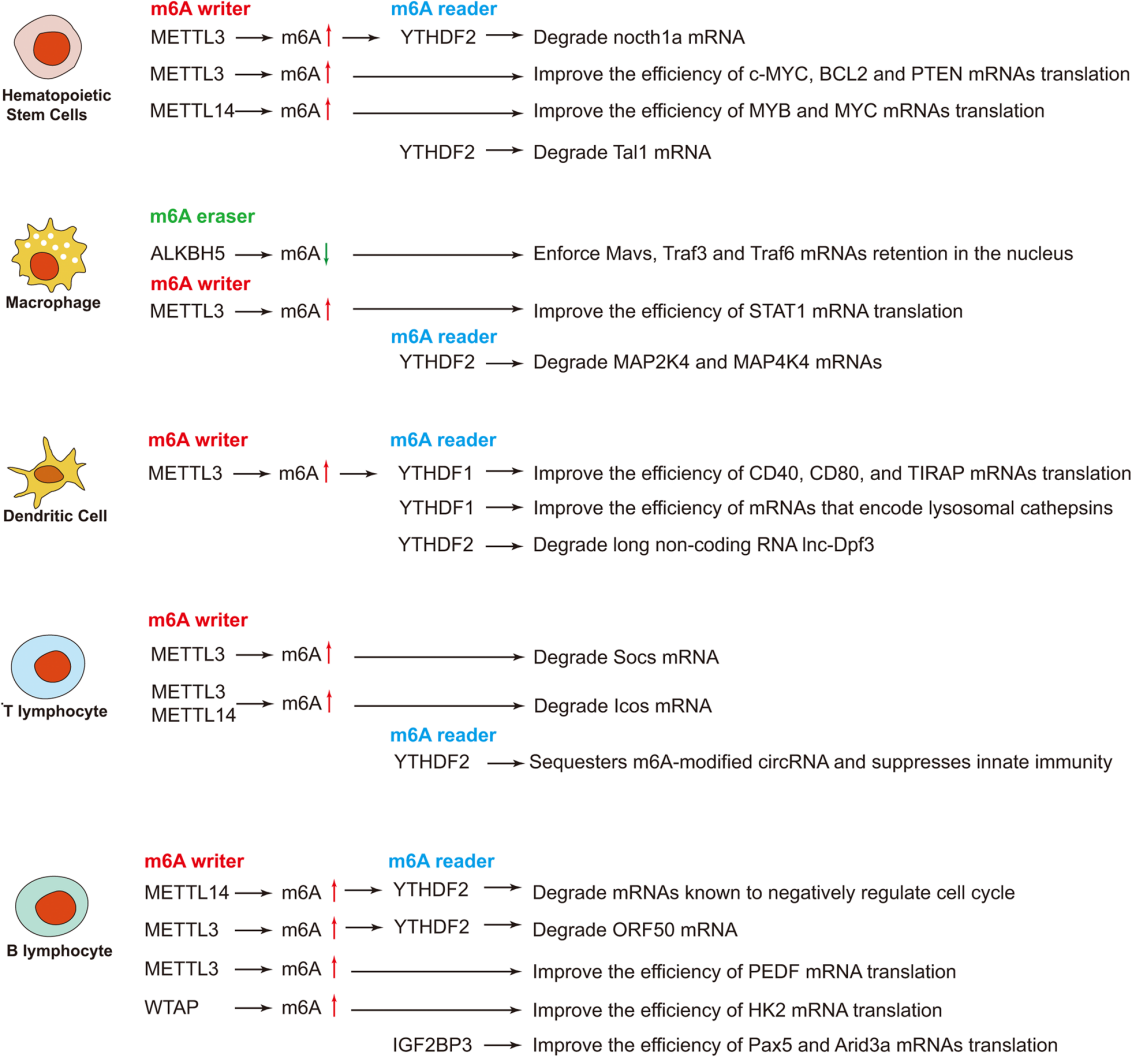
Detailed m6A regulatory pathways in immune cells that have been revealed. Only several m6A related proteins have been reported in the developments of HSCs, B cells and T cells

The blood cells originate from hematopoietic stem cells (HSC) in the bone marrow [67]. After released from the bone marrow, the progenitor cells can differentiate into various immune cells in blood under different combinations of cytokines and activating stimuli [68]. There are different types of immune cells, playing specific roles and establishing a strong crosstalk network in human innate immune system. As a burgeoning field of epigenetic regulation, RNA m6A modification has attracted more attention in the immune system. Although extensive research has been carried out solely on the regulatory roles of m6A in stem cells, seldom is revealed in the immune cells (Fig. 2). Therefore, the function and roles of m6A regulation needs to be further explored in most types of immune cells.

### Hematopoietic stem cells (HSCs)

HSCs are the source of various kinds of immune cells in blood, for these cells are capable of self-renewal. HSCs can differentiate into multiple lineages of blood cells, which is a tightly and highly regulated process [69]. The regulatory role of RNA m6A modification in HSC differentiation is emerging as the research focus because it possesses vital biological functions in different types of cells in either physiological or pathological process.

In early stages, the function of m6A regulation in hematopoietic stem and progenitor cells (HSPCs) was elucidated during zebrafish embryogenesis [70]. METTL3-mediated m6A regulation influences endothelial-to-hematopoietic transition (EHT) during the formation of HSPCs. And knockdown of METTL3 reduces both m6A level and cell number of HSPCs. It has been proven that m6A reader YTHDF2 promotes mRNA degradation and reduces the stability of m6A-modified transcripts. The delayed YTHDF2-mediated mRNA decay of genes *notch1a* and *rhoca* helps the activation of Notch signaling in arterial endothelial cells of *mettl3*-konckout embryos, which blocks the EHT and finally suppresses HSPCs generation. Moreover, knockdown of METTL3 in mice confers a similar phenotype [70]. In a separate study, researchers utilized the Cre/LoxP system to generate the endothelial-specific Mettl3 conditional knockout mice to identify the function of Mettl3-mediated m6A modification in mouse definitive hematopoiesis [71], which has proved the vital role of m6A modification in HSPCs formation during vertebrate embryogenesis. In human HSPCs, it is reported that the depletion of METTL3 promotes cell differentiation but reduces cell proliferation. In addition, the mRNA and protein levels of METTL3 are higher in acute myeloid leukemia (AML) cells than those in healthy HSPCs. METTL3 knock-down can also lead to cell differentiation and apoptosis in AML cell lines and delay disease progression in mouse leukemia models. The overexpression of METTL3 in AML cells results in the methylation of c-MYC, BCL2 and PTEN mRNAs, which leads to the maintenance of HSCs and the overexpression of BCL2 and PTEN. However, depletion of METTL3 down-regulates the expression of those genes and activates AKT, resulting in cell differentiation and apoptosis. The results suggest that m6A modification controls the differentiation of normal and/or malignant myeloid hematopoietic cells [72]. As the other m6A writer, METTL14 is highly expressed in normal HSPCs and various AML cells, with some functions differ from METTL3. METTL14 acts as an oncogene through regulating MYB and MYC mRNA and is down-regulated by SPI1 during myeloid differentiation [73]. Based on the vital roles of METTL3 and METTL14 discovered, METTL3 and METTL14 conditional knockout mice were established to elucidate their influence on HSC self-renewal in the bone marrow of adult mice [27]. The deletion of METTL3 with/without METTL14 in the hematopoietic system increases HSC frequency in the bone marrow; In contrast, deletion of METTL14 alone has little impact on the HSC self-renewal. These results proved that METTL3 is crucial for maintaining HSC quiescence and self-renewal potential [74]. Compared with METTL14, METTL3 is a dominator in m6A writer complex.

WTAP is identified as another important component of m6A methyltransferase complex. Proteomic analyses revealed that RNA binding motif protein 15 (RBM15) copurifies with WTAP [75]. And RBM15 has been confirmed to participate in m6A regulation by the study of the lncRNA XIST [38]. Interestingly, the important role of RBM15 in hematopoiesis had been confirmed before its role in m6A regulation was discovered. RBM15 is highly expressed in HSCs, and depletion of RBM15 increases the amount of HSPCs in mice. Besides these, RBM15 knockdown can blocks B cell differentiation [76], and suppress myeloid differentiation through the Notch signaling pathway [77]. Moreover, RBM15 is able to regulate HSCs and megakaryocyte development through c-Myc [78]. Though the vital role of RBM15 in HSCs has been explored, how RBM15 affects HSC differentiation via m6A regulation has not been reported yet.

YTHDF2 is proven to mediate mRNA decay through binding to m6A sites in mRNA. Previous research showed that following the conditional knockout of mouse YTHDF2 the numbers of functional HSC were increased without skewing lineage differentiation or leading to hematopoietic malignancies. Mechanically, YTHDF2 can degrade the m6A modified mRNAs which encode transcription factors critical for stem cell self-renewal, such as Tal1 mRNA [79].

### Macrophages

Macrophages are the potent immunoregulatory cells of the innate immune system involved in host defense against infections along with the maintenance of immune homeostasis or various pathological processes, including cancers [80]. As scavengers, macrophages remove worn-out cells and other debris. They are antigen presenting cells, that is why they have important function in immune response. In addition, as secretory cells, they can produce a series of cytokines and other regulatory factors. Therefore, macrophages are particularly necessary in immune response adjustment and the development of inflammation [20].

However, the role of m6A regulation in macrophages is rarely reported. It’s well known that DEAD-box (DDX) helicases are responsible for initiating innate antiviral immunity. One of nuclear DDX family members, DDX46 can inhibit the production of type I interferon after viral infection. DDX46 can bind to RNAs, such as Mavs, Traf3 and Traf6 transcripts, via their conserved CCGGUU element in macrophage nucleus. These transcripts encode signaling molecules which are vital for type I interferon production during antiviral responses. It has been shown that the m6A eraser ALKBH5 is involved in the antiviral responses of macrophages via m6A guided regulation. After viral infection, ALKBH5 is recruited by DDX46 through DDX46’s DEAD helicase domain to demethylate those m6A-modified antiviral transcripts. As a result, the transcripts without m6A modification will be retained in the nucleus, leading to the interferon production inhibition in the infected macrophages [81]. There are mainly two types of macrophages designated M1 and M2, which produce interferon γ (IFN-γ) with high bactericidal or pro-inflammatory activities and cytokine interleukin-4 (IL-4) with anti-inflammatory function respectively. The alterations in macrophage polarization between M1 and M2 are involved in various physiological and pathological processes. Recently, the role of m6A regulation during macrophage polarization has been investigated. It is reported that the m6A writer METTL3 is upregulated during M1 polarization of mouse macrophages by methylating STAT1 mRNA which is the important mediator of M1 macrophage polarization. The stability of m6A-modified STAT1 mRNA has been significantly increased and subsequently the STAT1 expression is upregulated. Thus, knockdown of METTL3 inhibits M1 but enhances M2 macrophage polarization. In the future, METTL3 may serve as a promising molecular target in anti-inflammatory treatments [82].

Lipopolysaccharide (LPS) is a major constituent in gram-negative bacteria cell wall. It stimulates macrophages to secrete different kinds of inflammatory cytokines, such as TNF-α, IL-6, IL-12, and IL-1β. The m6A reader YTHDF2 can recognize and bind to m6A modified sites on the mRNAs, influencing the stability of mRNAs. YTHDF2 depletion upregulates the expression and stability MAP2K4 and MAP4K4 mRNAs. Subsequently, the activation of MAPK and NF-κB signaling pathways leads to the expression of TNF-α, IL-6, IL-12, and IL-1β in LPS-stimulated macrophages.

### Dendritic cells (DCs)

As a “bridge” between the innate and the adaptive immune systems, DCs are antigen presenting cells that initiate an immune response by activating T cells [83]. The role of m6A in DCs has been illustrated before the abundant functions of m6A was discovered. It is well known that DNAs and RNAs can trigger the innate immune response via activation of toll-like receptors (TLRs) on DC surface. But when DCs are exposed to m6A-modified RNAs, the cytokines and activation markers has been significantly produced compared to those challenged by unmodified RNAs, suggesting that m6A modification impedes the DCs activation. Some researchers speculate that the presence of m6A on viral RNAs may help the virus avoid attacks from the host immune system. In addition, the innate immune system may detect RNAs lacking m6A as a means of selectively responding to bacteria or necrotic tissue [84]. Recently, it is reported that the loss of m6A reader YTHDF1 in DCs enhances antitumor immunity and inhibits tumor growth in mouse models. By recognizing and binding their target mRNAs, YTHDF1 enhances antigen degradation in DCs, which limits DCs to present tumor neoantigens to T cells. Therefore, YTHDF1 plays a key role in tumor immune evasion by regulating the activity of DCs. Furthermore, the therapeutic efficacy of PD-L1 checkpoint blockade is enhanced in YTHDF1 knock-out mice. In the near future, it may become a therapeutic target for improving the immune response against tumor [54].

CCR7 chemokine receptor stimulation induces rapid but transient DCs migration toward draining lymph nodes, which is essential for the initiation of protective immunity and maintenance of immune homeostasis [85]. As a CCR7 induced lncRNA, Lnc-Dpf3 can bind to HIF-1α and suppressed HIF-1α-dependent transcription of the glycolytic genes, resulting in the inhibition of CCR7-mediated DCs migration. In this research, Lnc-Dpf3 was found to consist of two active m6A sites within total 14 GGAC motifs. m6A-modified lnc-Dpf3 could be degraded when it’s recognized and bound by YTHDF2. Upon stimulation of CCR7, YTHDF2-mediated Lnc-Dpf3 degradation can be blocked by removing m6A modification. Moreover, the recognition of lnc-Dpf3 by YTHDF2 was also downregulated by CCR7 stimulation without changing YTHDF2 expression. The inhibition of DC migration via lnc-Dpf3 is important for the prevention of inflammatory pathogenesis and maintenance of immune balance [85].

The other study revealed that METTL3 can mediate the maturation and activation of DCs. CD40, CD80, and the TLR signaling adaptor protein (TIRAP) are crucial molecules in DCs for stimulating T cell activation. As a m6A writer, METTL3 can methylate their transcripts, and then YTHDF1 recognizes the m6A modified mRNAs of CD40, CD80, and TIRAP, increasing their translation to promote DC activation and DC-based T cell response [86].

### T cells and B cells

Lymphocytes arise from stem cells in bone marrow and differentiate in the central lymphoid organs. There are two types of lymphocytes critical for specific immune responses: T lymphocytes (T cells) and B lymphocytes (B cells). T cells mediate the cellular immune response, while B cells produce antibodies in humoral immune responses [87]. T and B cells are most important to the immune system, but the roles of m6A modification in T and B cells on their developments remain unclear.

### m6A modification in T cells

It has been proved that m6A methylation on mRNA controls T cell homeostasis. Some studies showed that the lower level of m6A (caused by Mettl3-KO) leads to decreased Socs mRNA degradation, resulting in the inactivation IL-7/STAT5/SOCS pathway. However, IL-7 stimulation activates the JAK/STAT pathway through m6A regulation which down-regulates expressions of SOCS family genes, initiating the re-programming of the naive T cells for differentiation and proliferation [88]. In addition, the researchers found that in regulatory T cells (Tregs) m6A regulates genes that encode components of vital signaling pathways for controlling naive T cells differentiation, and sustains the suppressive functions of Tregs. And it is possible that selective alteration of m6A in tumor-infiltrated Tregs may be useful for cancer immunotherapy [89].

With the help of bioinformatic analysis, researchers tried to quantify the RNA dynamics in T cells to uncover how these transcripts are regulated by m6A. In the regulation of T cells homeostasis, the loss of m6A reduces kinetic rates of RNA synthesis, processing, and degradation [90]. During HIV-1 infection of human CD4^+^ T cells, there is a massively increased level of m6A modification on both host and viral mRNAs, especially on the mRNAs critical for the virus. The m6A modification on viral mRNAs contributes to the binding of the Rev protein, which is essential for the exportation of the viral mRNAs from T cell nuclei [91]. While, the other study focuses on the m6A modification in circular RNAs (circRNAs) during the activation of antigen-specific T cells. circRNAs are the common RNAs formed in eukaryotic cells and viruses. The exogenous circRNAs induce the activation of antigen-specific T cell in vivo. Unmodified circRNAs activate RIG-I to cause filamentation of the adaptor protein MAVS and activation of downstream transcription factor IRF3, increasing interferon production. But the m6A-modified circRNAs will be sequestered by m6A reader YTHDF2, leading to suppression of the innate immunity [92].

Follicular helper T (Tfh) cells play an important role in the formation germinal centers (GCs) and mediates the effective humoral immunity. As the critical molecule for Tfh development, Icos expression is suppressed by the induced GAPDH protein through METTL3/METTL14-catalyzed m6A modification on Icos mRNA [93].

### m6A modification in B cells

m6A regulation is involved in several vital pathways controlling early B Cell development. In B cell precursors, Lin28b and IGF2BP3 are associated with the stability of mRNAs, such as B cell regulators Pax5 and Arid3a mRNAs [65]. Deletion of METTL14 severely impairs B cell development, which impacts the processes of IL-7-induced pro-B cell proliferation, large-pre-B-to-small-pre-B transition, and causes abnormalities in gene expression critical for B cell development [94]. Besides METTL14, YTHDF1 and YTHDF2 also function in different ways to influence early B cell development. Bioinformatic study showed that the expression levels of m6A regulators are regarded as identifiers in mantle cell lymphoma, a kind of aggressive B cell lymphoma. The low m6A level indicates a poor survival rate in patients with mantle cell lymphoma [95]. In diffuse large B-cell lymphoma (DLBCL), m6A level and METTL3 expression are increased both in tissues and cell lines. And the upregulated METTL3 promotes DLBCL cell proliferation by increasing the mRNA level of pigment epithelium-derived factor (PEDF) through m6A modification [96]. Moreover, another study showed that HK2 is a critical target gene of WTAP, which promotes the progression of DLBCL. And WTAP is upregulated by piRNA-30473 to enhance HK2 m6A level, promoting the expression of HK2. That is why piRNA-30473/WTAP/m6A/HK2 axis contributes to DLBCL tumorigenesis [97].

During virus infection, m6A also plays an essential role in the infected B cells. For example, one experimental study shows that m6A levels are significantly increased in the cells infected by the oncogenic human DNA virus Kaposi's sarcoma-associated herpesvirus (KSHV). In addition, reduced METTL3 and YTHDF2 significantly decrease virion production in KSHV infected B cells [98].

## Discussion and prospects

In recent years, the biological functions of m6A regulation are being explored and the number of researches related to m6A are obviously increased. In the beginning, most studies mainly focused on the physiological function of m6A modification in cells. As these normal functions have been clarified, the pathological roles and underlying molecular mechanism of m6A modification in different kinds of diseases are increasingly becoming a hot-trend research topic [99–101]. But the roles of m6A regulation in various immune cells are still not clear.

**Fig. 3:**
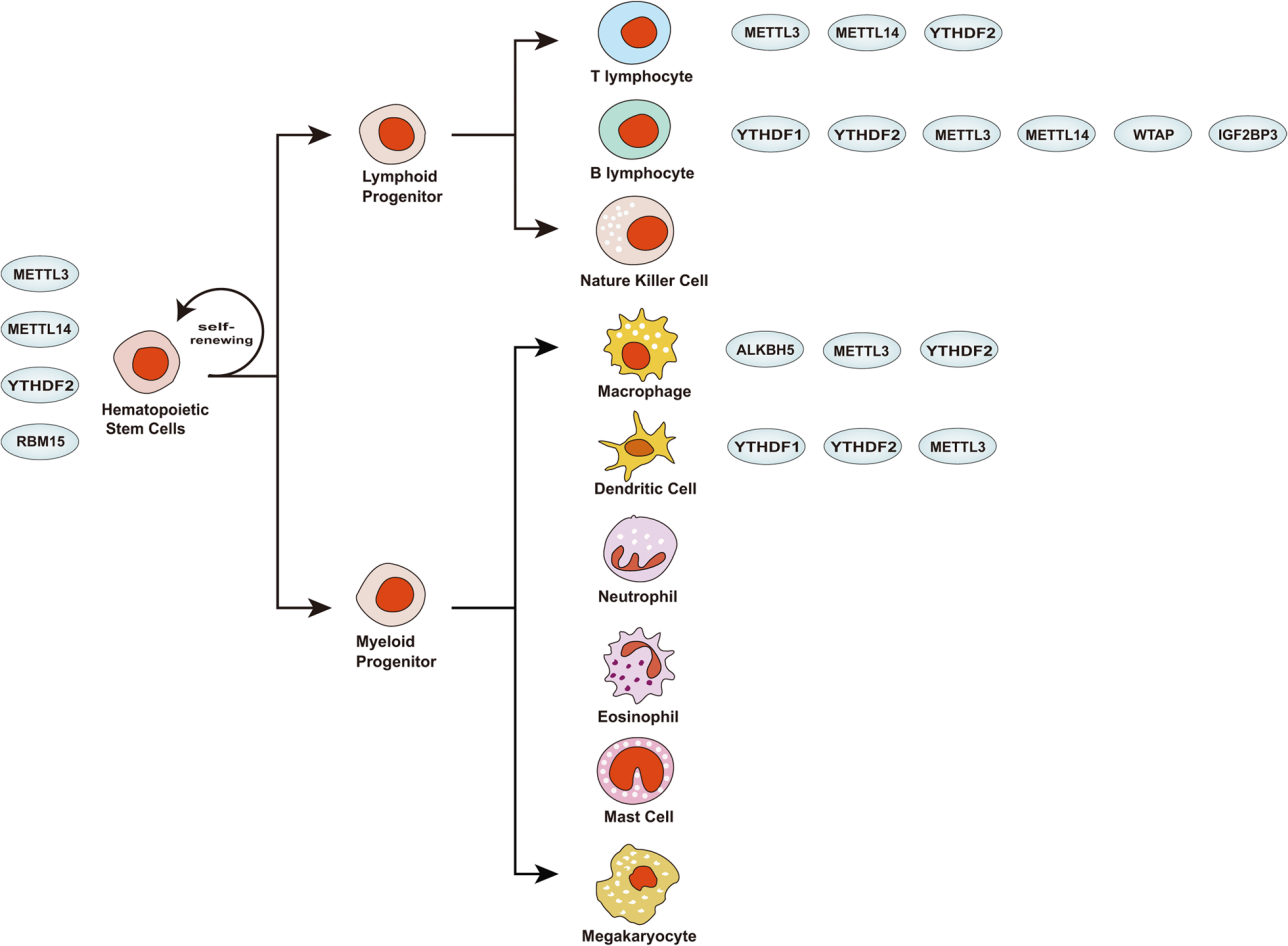
The m6A modification related proteins that are involved in the regulation of immune cells. METTL3, METTL14, RBM15 and YTHDF2 modulate the homeostasis of HSCs. METTL3, METTL14 and YTHDF2 participate in the regulation of T cells homeostasis. METTL3, METTL14, WTAP, YTHDF1, YTHDF2 and IGFBP3 have effects on the development of B cells. ALKBH5, METTL3 and YTHDF2 are involved in the polarization of macrophages. YTHDF1, YTHDF2 and METTL3 affects the activation of dendritic cells. However, m6A regulation in other immune cells have not been reported yet

**Fig. 4:**
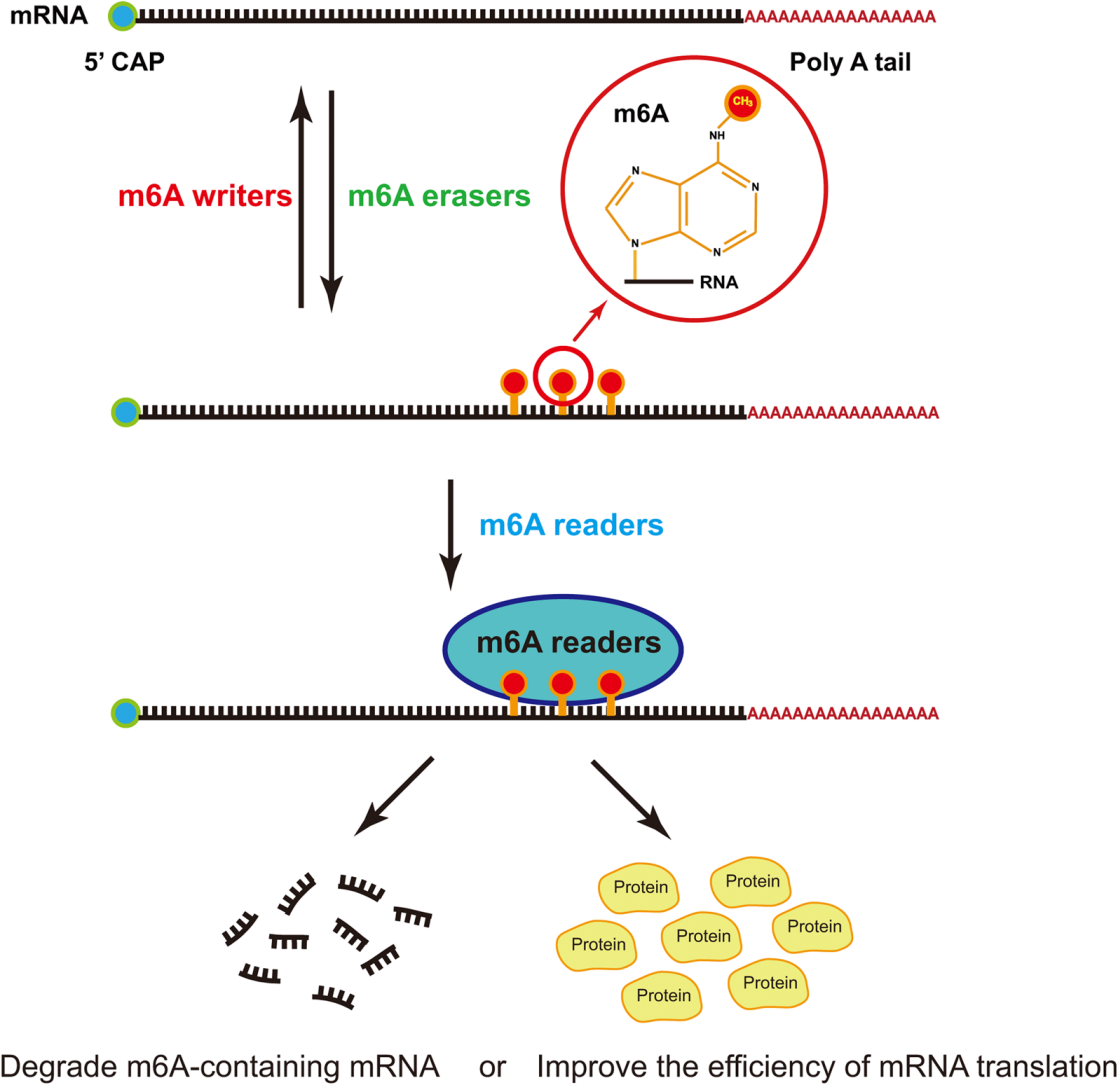
The m6A regulatory pathways have the similar axis of writer/eraser/m6A-reader/mRNA/cell functions in common. In general, writer or eraser proteins are responsible for adding or removing m6A modification, then reader proteins recognize the m6A-modified mRNAs, which are divided into different protein families to perform various functions in cells. The regulatory functions of m6A are usually mediated by this pathway in common.

In this review, we systematically summarize the members of m6A regulators (Fig. 3) and recent findings of m6A modification in some immune cells. And we also summarize the signal pathways in which m6A regulation is involved to seek their similarities. All m6A regulations are based on the addition or removal of methyl to or from RNAs done by writers/erasers. Then, the RNA methylation readers recognize and then degrade the m6A-modified mRNAs or promote their translation. The results of m6A regulation will be different due to the distinct functions of the modified mRNAs. Based on this review, it is not difficult to find that many regulatory pathways are similar with the axis of writer/eraser/m6A-reader/mRNA/cell functions [102–104] (Fig. 4). The difference lies in that different m6A-related regulatory proteins are involved in different physiological or pathological alterations, which suggests that we need to analyze specific cases respectively [105, 106].

This review also tries to point out the regulatory role of m6A modification in various immune cells. However, the m6A-related researches are not carried out and reported in all immune cells. This review is conceived on the literature that has been reported. The immune cells not listed in this review are because no m6A studies have been reported in them. Nevertheless, it does not mean that m6A has no effects in these cells. This has a great suggestive effect and reminds researchers of the roles of m6A in more immune cells. Nowadays, there are more studies on the m6A modifications in HSCs only, therefore, what functions m6A plays in immune cells will be of great interest and worth further exploration.

There are some challenges in the field of m6A research. More techniques have been applied to m6A sequencing, but the specific modification sites determined may have certain deviations. Moreover, m6A modification is a dynamic and reversible process, so how to accurately find the key target mRNAs that are regulated by m6A is still a major challenge. In addition, m6A regulation is of cell heterogeneity, that is, the same writer, eraser, and reader proteins may have different biological functions in different cells. This is particularly significant in the field of cancer research, for example, YTHDF1 can play both a cancer-promoting and a cancer suppressing role in different cancers. Therefore, it is also necessary to pay attention to this point in the research of immune cells. There may be large differences in the m6A functions due to the different types of immune cells. Furthermore, when and how writer, eraser and reader proteins are involved in the m6A regulation, and by what factors are these proteins affected, remain unresolved at present. We think the specific opportunity when m6A participates in regulation of cell functions will be further clarified soon.

## Data Availability

All datasets used or analyzed during the current study are available from the corresponding author on reasonable request.
